# Prevalence of linezolid-resistant organisms among patients admitted to a tertiary hospital for critical care or dialysis

**DOI:** 10.1007/s11845-021-02773-2

**Published:** 2021-09-10

**Authors:** Kornelia Maria Dembicka, James Powell, Nuala H. O’Connell, Noreen Hennessy, Grainne Brennan, Colum P. Dunne

**Affiliations:** 1 Department of Clinical Microbiology, University Hospital Limerick, Limerick, Ireland; 2 Centre for Interventions in Infection, Inflammation & Immunity (4I) and School of Medicine, University of Limerick, Limerick, Ireland; 3 Public Health Laboratory, Raheen Business Park, Limerick, Ireland; 4 National MRSA Reference Laboratory, St James’s Hospital, Dublin, Ireland

**Keywords:** Colonization, Linezolid, Resistance, Screening, *Staphylococcus epidermidis*

## Abstract

**Background:**

Linezolid is an oxazolidinone antimicrobial regarded as a “last resort” antimicrobial, used typically for treatment of Gram-positive bacterial infections. It is acknowledged that prevalence of resistance to linezolid is increasing in Europe. In Ireland, a number of outbreaks of linezolid-resistant isolates have been reported, including an outbreak at the location for this study, the Intensive Care Unit (ICU) of University Hospital Limerick (UHL).

**Methods:**

The Chromagar™ Lin-R selective medium was validated using a panel of linezolid-sensitive and linezolid-resistant strains. Subsequently, the prevalence exercise focused on a convenience sample of patients (*n* = 159) in critical care wards, ICU (*n* = 23) and High-Dependency Unit (HDU, *n* = 51), in addition to patients undergoing dialysis therapy (*n* = 77). Eight additional patients had specimens collected when attending more than one location. Growth on Chromagar™ Lin-R agar was followed by drug sensitivity testing by disc diffusion and minimum inhibitory concentration (MIC) testing.

**Results:**

A validation exercise was performed on 23 isolates: seven target and sixteen non-target organisms. Isolates performed as intended (100% sensitivity, 100% specificity). For the prevalence study, of 398 tests, 40 resulted in growth of non-target organisms (specificity approx. 90%). A sole patient (1/159) was identified as colonized by a linezolid-resistant *Staphylococcus epidermidis*, a prevalence of 0.63%. Molecular investigation confirmed presence of the G2576T mutation in the 23S rRNA.

**Conclusion:**

While this point prevalence study identified extremely low carriage of linezolid-resistant bacteria, it remains prudent to maintain vigilance as reports of outbreaks associated with linezolid-resistant *S. epidermidis* (LRSE) in European critical care units are increasing.

## Introduction

Linezolid was the first oxazolidinone antimicrobial agent approved by the FDA, in 2000. Its mode of action is the inhibition of synthesis of bacterial proteins through interaction with both 30S and 50S ribosomal subunits [1]. More specifically, linezolid inhibits production of virulence factors in *Staphylococcus aureus* and *Streptococcus pyogenes* [2] with 5-acylaminomethyl and N-aryl groups implicated in this mechanism [1]. Relatively recently, linezolid was recommended for the treatment of multi-drug-resistant tuberculosis infections [3, 4]. However, in the Republic of Ireland, the antimicrobial is currently approved for treatment against nosocomial and community-acquired infections caused by multi-drug-resistant Gram-positive organisms, including pneumonia, and soft-tissue and skin infections. It remains the antimicrobial of choice from the reserve group in Irish facilities, a medication of “last-resort”, as fortunately the majority of Gram-positive bacterial pathogens remain susceptible [5]. Internationally, linezolid resistance has been reported, correlating with mutations of the L3, L4 and the L22 ribosomal proteins or a mutation of the 23S ribosomal RNA, G2576T [6]. Resistance has also been associated with horizontally transferred genes including *cfr, cfr(B), poxtA* and *optrA* [6].

Reference laboratories across Europe have reported increased incidence of linezolid-resistant isolates, including Germany where prevalence of linezolid-resistant enterococci rose from < 1% to > 9% between 2008 and 2014 [7]. In Ireland, linezolid-resistant *S. epidermidis* isolates, exhibiting the G2576T mutation, have been identified as cause of the first reported outbreak in The Adelaide and Meath Hospital Incorporating the National Children's Hospital (AMNCH), Dublin, which ran its course between 2005 and 2006 [8]. In our hospital, University Hospital Limerick (UHL), a linezolid-resistant *S. aureus* harbouring the G2576T mutation was isolated from a cystic fibrosis patient in 2005 [9] while we reported the first *cfr*-mediated linezolid-resistant *S. epidermidis* outbreak in an Irish Intensive Care Unit (ICU), involving nine colonized or infected patients in 2013 [3]. A study by Egan et al*.* [10] revealed enterococci in Irish hospitals exhibited high transmissibility, implicating *optrA* and *poxtA* genes. Subsequent to these reports, the Irish Health Protection Surveillance Centre recommended screening of individuals who are at risk for carriage of antimicrobial-resistant organisms [11] upon presentation at hospitals.

Patients in critical care units are particularly at risk of developing infections with drug-resistant organisms. The majority of these patients are treated empirically with broad-spectrum antibiotics on admission as antibiotic conservation in such acute settings is difficult to employ [12]. Perhaps unsurprisingly, outbreaks of drug-resistant organisms often begin in critical care units. Such was the case for the first linezolid-resistant *S. epidermidis* (LRSE) outbreak reported in Ireland, which began in an intensive therapy unit [8]. Two further outbreaks confined to ICU units of LRSE have been reported in the Republic of Ireland, emphasising the necessity for surveillance of linezolid-resistant organisms in this cohort of patients [3, 13].

In that context, new screening and diagnostic innovations are attractive. However, in each case, they require appraisal and evaluation relevant to the location where they may be implemented. Therefore, our objective was to evaluate a novel chromogenic agar, Chromagar™ Lin-R [14]. This newly developed medium, previously validated for use in Germany with 100% specificity and 99% sensitivity described [15], may aid economically viable routine screening. To perform this evaluation, we chose a critical care and dialysis patient setting as incidence of extended-spectrum beta-lactamase-producing organisms colonizing community patients was studied previously at UHL, demonstrating the feasibility of conducting a linezolid-resistance prevalence study [16]. There have been no studies published to date that investigated the prevalence of linezolid resistance among patients in critical care and dialysis units in Irish hospitals.

## Materials and methods

### Setting

University of Limerick Hospitals Group (ULHG) comprises UHL, University Maternity Hospital Limerick, Nenagh Hospital, Ennis Hospital, Croom Hospital and St. John’s Hospital. The ICU and HDU represent 24 in-patient beds. The ICU accommodates critically unwell patients and differs from HDU by nurse per patient ratio. The dialysis unit in UHL has five bays, which can cater for 20 patients at any time and 4 isolation rooms. Patients undergoing treatment in ICU, HDU and dialysis units were included in this study. The preparation of the Chromagar™ Lin-R agar plates and their validation was performed in the affiliated Public Health Laboratory and UHL Microbiology Department.

### Preparation of chromagar™ lin-r agar

Chromagar™ Lin-R chromogenic medium [14] comprises powder base and supplement. Preparation of Chromagar™ Lin-R agar is as follows: 42.4 g/L of powder base, which was composed of chromogenic and selective mix (0.4 g/L), salts (7 g/L), agar (15 g/L) and peptones (2.2 g/L). The supplement is a proprietary growth factor, and a total of 8 mL/L was added to the mixture prior to autoclaving at 110 °C for 5 min. The plates were stored in the dark at 2–8 °C for the duration of the study. Three control strains were utilized, *S. epidermidis* NCTC 13,360, *S. aureus* NCTC 12,493 and *Enterococcus faecalis* NCTC 13,379.

### Validation of chromagar™ lin-r agar

**Fig. 1:**
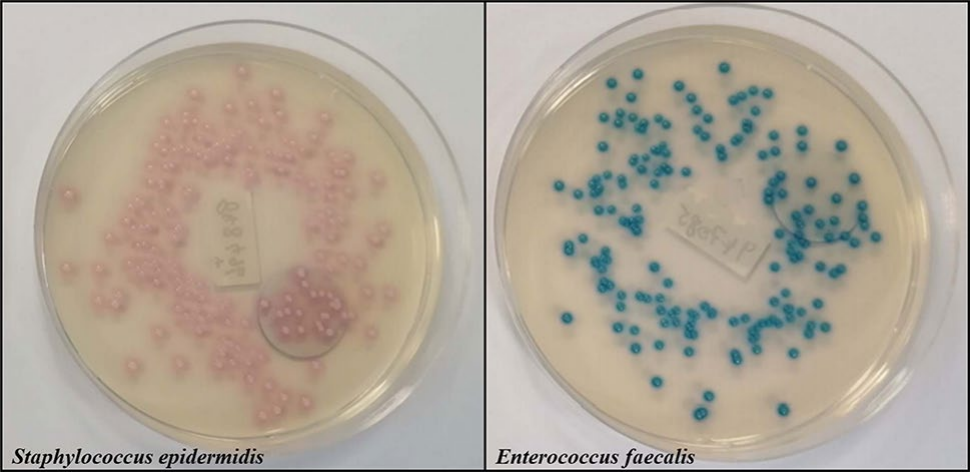
Linezolid-resistant *Staphylococcus* species on the chromogenic medium Chromagar™ Lin-R agar have pink typical appearance, in contrast to *Enterococcus* species, which are steel blue in colour

The chromogenic and selective properties of the agar were evaluated prior to commencement of the surveillance study. A total of 28 isolates were employed, seven linezolid-resistant organisms and 21 non-target organisms (Gram-positive isolates susceptible to linezolid, Gram-negative isolates and a yeast). Linezolid-resistant *Enterococcus* species had a steel blue typical colony appearance, in contrast to the pink colour of linezolid-resistant *Staphylococcus* species (Fig. 1). All seven target (linezolid-resistant) isolates were readily identifiable on the chromogenic agar after 18–24-h incubation (100% sensitive), and all 21 non-target organisms were successfully inhibited (100% specific).

### Sample collection

**Fig. 2:**
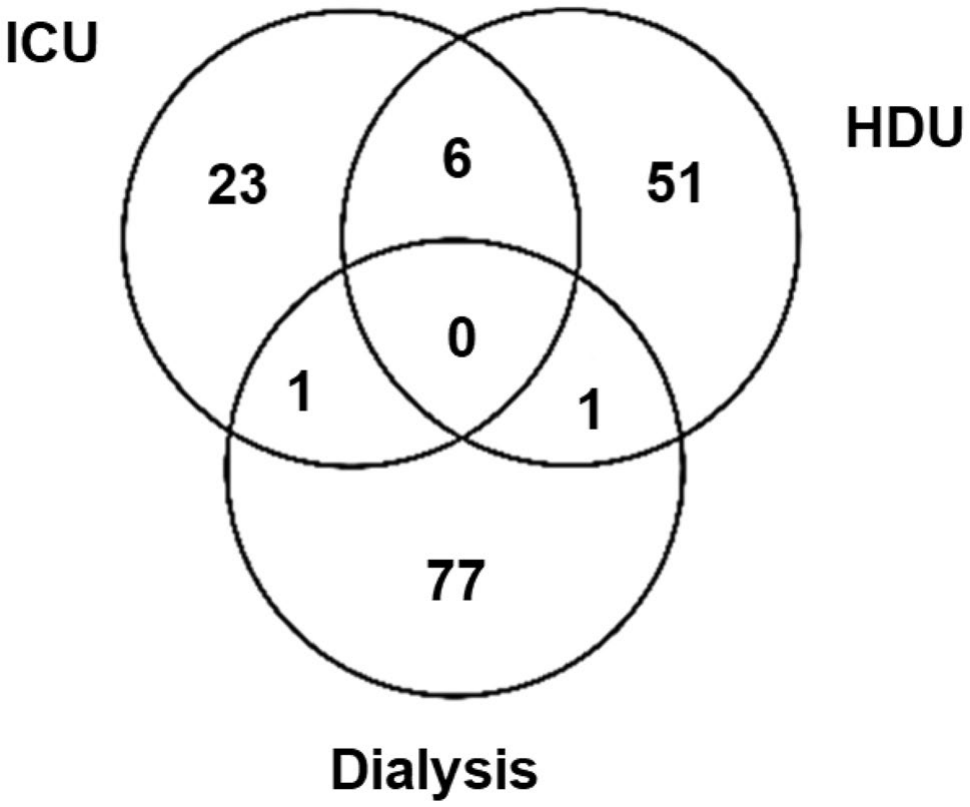
159 patients were sampled at these treatment locations

**Fig. 3:**
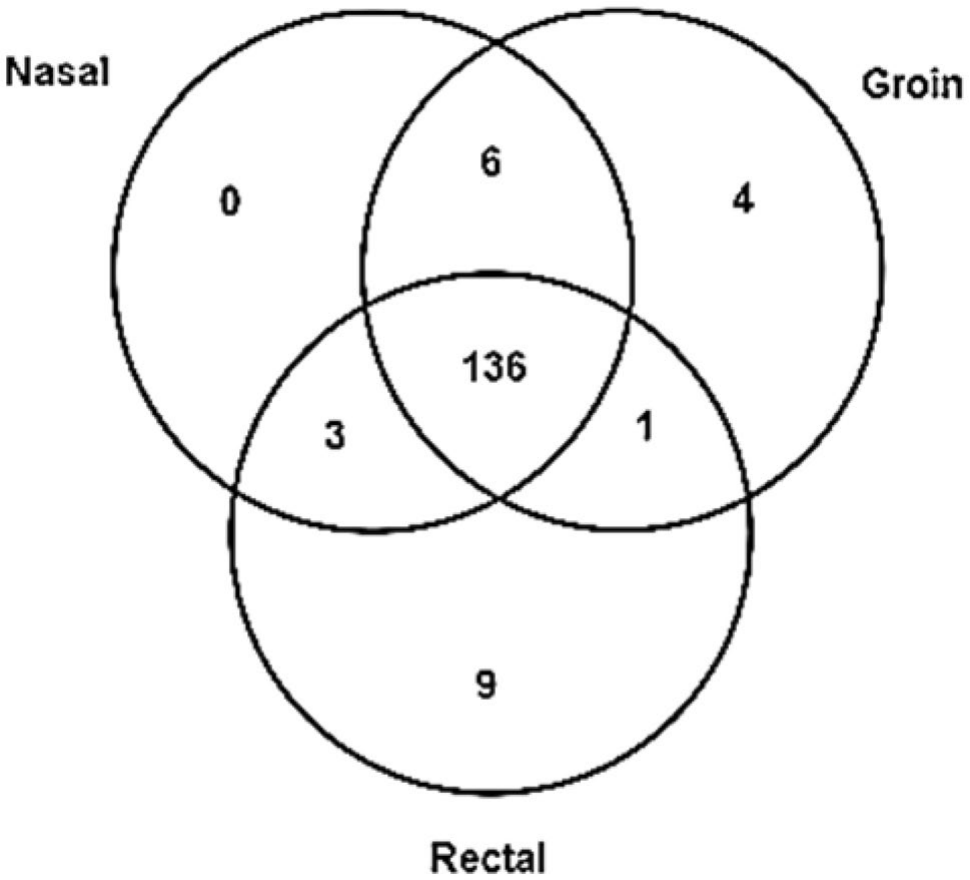
159 patients provided swabs from one of more of these physiological sites

Patients in the critical care units, ICU and HDU, in addition to dialysis care patients were targeted for the purpose of this study. The ICU and HDU patients are screened for meticillin-resistant *S. aureus* (MRSA) and vancomycin-resistant enterococci (VRE) utilizing Amies charcoal transport swabs (“charcoal swabs”, Deltalab, Spain) on admission, weekly thereafter and on discharge. Patients undergoing dialysis treatment are routinely screened quarterly. Exclusion criteria included incorrectly labelled swab, less than two identifiers corresponding to requesting form, incorrect specimen containers and swabs taken from body sites not under investigation. Charcoal swabs were stored at room temperature and tested within 48 h of collection. One hundred fifty-nine patients were screened for the presence of linezolid-resistant organisms (Fig. 2), with some patients having samples collected from multiple locations. The patients’ ages ranged from 16 to 92 years (M = 64.4, SD = 16.4), with 58% identified as male. The mean age of the dialysis patients was higher (67.1 years, SD = 14.7 years) and a greater preponderance of males (67%). The mean ages of the ICU and HDU patients were lower (M = 59.1 and 63.4; SD = 16.9 and 17.6 respectively), and they consisted of 60% and 45% males respectively. The majority of patients (86%, 136/159) were swabbed at all three body sites (nasal, groin and rectal) on at least one occasion, while the remaining patients were sampled from one or two of the three sites (Fig. 3).

A total of 398 samples were collected comprising 15 obtained while in the dialysis unit, 138 specimens at the private dialysis centre, 141 collected in HDU and 104 taken in ICU. Patient details were recorded on the laboratory information system (iLAB, DXC Technology).

### Screening for linezolid resistance and identification of isolates

Charcoal swabs were cultured directly on prepared Chromagar™ Lin-R agar. Nasal and groin swabs collected on the same day were cultured directly on the same agar plates. The rectal swabs were cultured onto individual agar plates. Following 18–24-h incubation, plates were examined for growth and then re-incubated for further 24 h. No further investigation was conducted if, after 48 h, no growth was observed. If growth was evident on the Chromagar™ Lin-R agar, colonies were cultured again onto chromogenic Chromagar™ Lin-R agar, and also onto McConkey agar without salt, Columbia Blood agar and Staph/Strep agar (LIP Diagnostics, Fannin Healthcare). Plates were incubated for 18–24 h at 35–37 °C. The sub-cultured isolates were further identified via Gram Stain and/or Matrix-assisted laser desorption/ionization-time of flight (MALDI-TOF) mass spectrometry (Bruker). Oxidase test (Serosep, Limerick, Ireland) was performed on organisms exhibiting typical colony appearance for *Pseudomonas* species.

### Confirmation of linezolid resistance

Sensitivity testing was performed utilizing Mueller Hinton agar plates (LIP Diagnostics, Fannin Healthcare) and following EUCAST breakpoint guidelines [17]. Any organisms demonstrating reduced susceptibility via the disc diffusion test were further investigated for their linezolid MIC utilizing the bioMérieux linezolid ETEST. MIC breakpoint values were obtained from EUCAST guidelines where available. Organisms that failed to grow on Mueller Hinton agar for sensitivity testing or were found to exhibit resistance to linezolid were referred to the Irish National MRSA Reference Laboratory (NMRSARL) in St. James’s Hospital, Dublin, for molecular confirmation of resistance genes as previously described [18].

## Results

In total, 398 samples recovered from 159 patients were screened for the presence of linezolid-resistant organisms using the Chromagar™ Lin-R agar, between 8th February and 3rd March 2021. Of those, 89% (354/398) yielded no growth. Eight specimens (2% of the total, four nasal/groin swabs and four rectal swabs) yielded a target linezolid-resistant organism, all *S. epidermidis* and all from the same patient. The linezolid MIC of this isolate was > 256 mg/L. The detection of one positive case among 159 patients tested represents prevalence of 0.96% for ICU, 0% for HDU and 0% for each dialysis unit, amounting to total prevalence of 0.63%. This patient was detected upon admission, having transferred from another hospital outside our region. The isolate was shown to lack the transferable *cfr*, *optrA* and *poxtA* genes, while the G2576T mutation was detected.

Four of the above positive specimens involved a mixed culture which included non-target organisms, and another thirty-six specimens also yielded non-target organisms, in total 40 specimens or approximately 10% of the total. These 40 tests yielded 50 non-target organisms including *Candida* (*n* = 1), Gram-negative organisms (*n* = 8) and linezolid-susceptible Gram-positive organisms (*n* = 41) (see Table 1). The high number of linezolid-susceptible organisms isolated can be attributed to our decision to investigate all growth on the chromogenic agar, even if the growth was partially inhibited by the selective properties of the agar. This approach was undertaken in order to maximize the sensitivity of the agar as a screening tool.

**Table 1:** Summary of non-target organisms isolated on the Chromagar™ Lin-R agar (i.e. Gram-negative organisms, Gram-positive organisms susceptible to linezolid and Candida species)

**Organism**	**Genus + / − species**	**Number of isolates**
**Gram-positive,** **linezolid susceptible**	*Enterococcus faecalis*	15
	*Enterococcus faecium*	14
	*Lactococcus* species	4
	*Enterococcus avium*	1
	*Enterococcus casseliflavus*	1
	*S. aureus*	1
	*S. simulans*	1
	*Weisella confusa*	1
	Not identified	3
**Gram-negative**		8
**Candida**		1
**Total**		**50**

## Discussion and conclusion

Linezolid is an effective antimicrobial against Gram-positive bacteria, and despite its comprehensive use for almost two decades, the 2014 LEADER surveillance programme in the USA determined linezolid’s sustained susceptibility rate as > 99.78% [5]. Unfortunately, as previously stated, reference laboratories in Europe have recorded a rise in prevalence of linezolid-resistant organisms [7]. This reflects increased incidence of antimicrobial resistance [19]. And, perhaps unsurprisingly, a correlation with relatively elevated numbers of ICU admissions and associated mortality rates [20]. In Ireland, a number of outbreaks involving linezolid-resistance have been reported, a significant proportion of which originated in ICU wards [8, 13].

Surveillance of in-patient colonization by resistant organisms is an effective infection control approach, as demonstrated by the exceptionally low prevalence of MRSA (approx. 1.7%) in The Netherlands. The Dutch “Search and Destroy” surveillance policy is thought to be responsible for this low rate, and its benefits include reduced mortality rate associated with *S. aureus* infection [21]. The objective of this study was to evaluate Chromagar™ Lin-R agar and to perform a point prevalence study of linezolid-resistant organisms on selected critical care patients.

The initial validation study demonstrated the efficacy of the Chromagar™ Lin-R agar as a screening tool (i.e. all target organisms grew readily and with appropriate pigmentation, while all non-target organisms were fully inhibited). Therefore, the medium was deemed appropriate as the screening method for the subsequent surveillance study. However, non-target organisms were not as successfully inhibited in the real-world analysis of patient specimens, with linezolid-susceptible Gram-positive organisms including *E. faecalis* and *E. faecium*, Gram-negative species and Candida isolated during the prevalence study. A total of 50 non-target organisms were identified from 40 out of 398 tests, representing approximately 90% specificity, contrasting with 100% specificity in our validation trial and in a validation study published previously [15]. However, in both cases the tests were performed with bacterial suspensions of single isolates, approximately 1.5 × 10^6^ CFU. In contrast, our study used swabs of heavily colonized body sites. Many of the non-target organisms were readily dismissed (e.g. using Gram stain), and this chromogenic agar has only recently been developed and, thus, it is still unfamiliar to scientists. Furthermore, the specificity of the agar was affected through our choice of pursuing all evident growth in order to maximize sensitivity. With greater experience of the colonial morphology and pigmentation of these non-target organisms on Chromagar™ Lin-R, it is likely that in-use specificity would increase. Overall, it is our view that Chromagar™ Lin-R agar is suitable as a screening tool for linezolid-resistant organisms as similar platforms are employed widely for MRSA and VRE.

*S. epidermidis* has been recognized as an organism that is particularly prone to development of linezolid resistance. For example, in Greece, linezolid resistance was established in 20.9% of *S. epidermidis* isolates recovered from ICU patients in 2013 [22]. Similar prevalence was reported for an ICU in Spain, where the increase of the rate was not hindered through implementation of isolation and control protocols [23]. Despite increased awareness, in 2020 the recovery of LRSE in ten patients treated in a children’s hospital in Poland (where linezolid was frequently used for severe infections) resulted in calls for enhanced surveillance and more stringent infection control policies [24]. In UHL, a 2013 outbreak of LRSE, mediated through the horizontally transferrable gene “*cfr*”, led to adoption of education programmes aimed at limiting the transmissibility of drug-resistant organisms and increased control in the prescription of linezolid [3]. In this current study, only one patient (1/159) was found to be colonized by a linezolid-resistant *S. epidermidis*. Prior to beginning treatment in UHL, the patient had received antimicrobial therapy including linezolid. In light of this, it is reasonable to propose that the procedures implemented in 2013 continue to be effective in our hospital.

In conclusion, the low prevalence (< 1%) of linezolid-resistant organisms in UHL observed in this surveillance study is reassuring. Nonetheless, as demonstrated by outbreaks and prevalence of LRSE in Europe, the colonization by LRSE of a single patient can be viewed as an opportunity to further enhance the protocols in place. Although the introduction of a linezolid screening schedule may not be appropriate due to the low prevalence observed, introduction of mandatory linezolid susceptibility testing of all *S. epidermidis* clinical isolates from critical care and dialysis wards could have potential to hinder future outbreaks and improve patient care.

## Data Availability

Raw data are available on request.
